# Change of Computed Tomography-Based Body Composition after Adrenalectomy in Patients with Pheochromocytoma

**DOI:** 10.3390/cancers14081967

**Published:** 2022-04-13

**Authors:** Yousun Ko, Heeryoel Jeong, Seungwoo Khang, Jeongjin Lee, Kyung Won Kim, Beom-Jun Kim

**Affiliations:** 1 Biomedical Research Center, Asan Institute for Life Sciences, Asan Medical Center, Seoul 05505, Korea; yousunko@amc.seoul.kr; 2 School of Computer Science and Engineering, Soongsil University, Seoul 06978, Korea; heeya8876@soongsil.ac.kr (H.J.); swkhang@soongsil.ac.kr (S.K.); leejeongjin@ssu.ac.kr (J.L.); 3 Department of Radiology and Research Institute of Radiology, Asan Medical Center, University of Ulsan College of Medicine, Seoul 05505, Korea; 4 Division of Endocrinology and Metabolism, Asan Medical Center, University of Ulsan College of Medicine, Seoul 05505, Korea

**Keywords:** pheochromocytoma, obesity, sarcopenia, body composition, catecholamine excess

## Abstract

**Simple Summary:**

Pheochromocytoma is regarded as a good human model for investigating the impact of sympathetic hyperactivity on various pathologic conditions. To determine the influence of catecholamine excess on human body composition, we compared computed tomography (CT)-based fat and skeletal muscle indices over time in a large patient population with histologically confirmed pheochromocytoma who underwent surgery. We observed considerable elevation in CT-measured visceral fat area and subcutaneous fat area, and the prevalence of visceral obesity after adrenalectomy in patients with pheochromocytoma. In contrast, there were no changes in skeletal muscle area, skeletal muscle index, and prevalence of sarcopenia. Furthermore, we observed that the severity of catecholamine excess was associated with a higher increase, especially in the subcutaneous fat area after surgery. These data provide important clinical evidence that sympathetic hyperactivity contributes to lipolysis in visceral and subcutaneous adipose tissues, whereas its impact on human skeletal muscle remains undetermined.

**Abstract:**

Despite the potential biological importance of the sympathetic nervous system on fat and skeletal muscle metabolism in animal and in vitro studies, its relevance in humans remains undetermined. To clarify the influence of catecholamine excess on human body composition, we performed a retrospective longitudinal cohort study including 313 consecutive patients with histologically confirmed pheochromocytoma who underwent repeat abdominal computed tomography (CT) scans before and after adrenalectomy. Changes in CT-determined visceral fat area (VFA), subcutaneous fat area (SFA), skeletal muscle area (SMA), and skeletal muscle index (SMI) were measured at the level of the third lumbar vertebra. The mean age of all patients was 50.6 ± 13.6 years, and 171/313 (54.6%) were women. The median follow-up duration for repeat CTs was 25.0 months. VFA and SFA were 14.5% and 15.8% higher, respectively (both *p* < 0.001), after adrenalectomy, whereas SMA and SMI remained unchanged. Similarly, patients with visceral obesity significantly increased from 103 (32.9%) at baseline to 138 (44.1%) following surgery (*p* < 0.001); however, the prevalence of sarcopenia was unchanged. This study provides important clinical evidence that sympathetic hyperactivity can contribute to lipolysis in visceral and subcutaneous adipose tissues, but its impact on human skeletal muscle is unclear.

## 1. Introduction

Pheochromocytoma and paraganglioma are catecholamine-secreting neuroendocrine tumors arising from chromaffin cells of the adrenal medulla and sympathetic paravertebral ganglia, respectively [1]. In addition to the classic triad of symptoms including episodic palpitations, sweating, and headache, catecholamine overproduction is responsible for many cardiovascular and metabolic alterations in patients with pheochromocytoma [2]. For example, a recent study reported that elevated cholesterol levels were improved by surgical resection in patients with pheochromocytoma [3]. Catecholamines are key neurotransmitters of the sympathetic nervous system (SNS), and they exert their biological effects through adrenergic receptors (ARs). Pheochromocytoma is therefore regarded as a good human model for investigating the impact of sympathetic hyperactivity on various pathologic conditions [4,5].

In an era wherein sedentary lifestyles and high-fat diets are common, obesity and sarcopenia are becoming increasingly relevant to public health [6,7,8]; thus, research on critical modulators affecting body composition is actively pursued. Interestingly, adipose tissue and skeletal muscle express abundant ARs [9,10,11,12], and accumulating evidence from in vitro and animal studies indicates clear implications for SNS in fat and muscle metabolism [12,13,14,15]. Therefore, research has been conducted to examine the effects of catecholamine excess on body composition in patients with pheochromocytoma. However, these studies did not consider fat and muscle together and had various limitations, including small sample sizes and cross-sectional design [16,17,18,19]. Large-scale longitudinal investigations are thus required to thoroughly elucidate the causal consequences of sympathetic overstimulation on body composition in humans.

A single-slice computed tomography (CT) scan at the level of the third lumbar vertebra (L3) is a well-established tool to assess the tissue volumes of subcutaneous and visceral fat and skeletal muscle [20,21,22]. Moreover, a fully convolutional network-based segmentation system of CT images has greatly improved its accuracy and reproducibility [23]. Importantly, unless contraindicated, all patients with pheochromocytoma should undergo adrenalectomy or mass excision, and abdomen CT should be taken before and after surgery to screen for metastasis and recurrence, respectively. Therefore, to determine the influence of SNS on human body composition, we compared CT-based fat and skeletal muscle indices over time in a large patient population with pheochromocytoma who underwent surgery and investigated the associations of catecholamine metabolites with these parameters.

## 2. Materials and Methods

### 2.1. Study Participants

Between January 2005 and December 2019, 441 patients were histologically confirmed with pheochromocytoma or paraganglioma among those who underwent adrenalectomy or mass excision at the Asan Medical Center ((AMC) Seoul, Korea). Of these, 313 patients had abdominal CT scans before and 3 to 48 months after surgery and were subsequently included in this study. This study was approved by the Institutional Review Board of AMC (No. 2021-0208), and the requirement for patient informed consent was waived given the retrospective nature of the study. Data were obtained from the Clinical Data Repository system, electronic medical records, and the radiology picture archiving and communication system at AMC.

### 2.2. Laboratory Measurements

Plasma fractionated metanephrine levels were measured by liquid chromatography–tandem mass spectrometry analysis using a Xevo-TQs tandem mass spectrometer (Waters Corporation, Milford, MA, USA) and an ACQUITY UPLC column (2.1 × 50 mm BEH Amide 1.7 μm; Waters Corporation). The limit of detection and quantitation were 0.06 nmol/L and 0.08 nmol/L, respectively. The reference ranges for plasma metanephrine and normetanephrine were less than 0.5 nmol/L and 0.9 nmol/L, respectively. Twenty-four-hour urine fractionated metanephrine levels were measured with an HPLC assay using a commercially available kit (Chromsystems, Munich, Germany) on an Agilent 1100 HPLC System (Agilent Technologies, Santa Clara, CA, USA). The lower limit of detection for the kit was 5–11 μg/L, and the intra- and inter-assay CVs were <3.0% and <4.4%, respectively. The reference ranges for urine metanephrine and normetanephrine were less than 341 μg/day and 444 μg/day, respectively.

### 2.3. CT Image Acquisition

All CTs were performed using a 16-channel or higher (LightSpeed VCT and Discovery CT 750 HD, GE Healthcare, Milwaukee, WI, USA; Somatom Definition AS+, and Somatom Definition Edge, Siemens Medical Solution, Erlangen, Germany) CT scanner with the following parameters: tube voltage, 120 kVp; effective tube current, 50–400 mA (AutomA or SmartmA; GE Healthcare) or 200 reference mAs (care dose 4D; Siemens Medical Solution); field of view, 30–40 cm; section thickness, 5 mm. Contrast agents were administered at a rate of 3–4 mL/s, and CT images were obtained, including the portal venous phase (120 s after contrast agent injection) in the craniocaudal direction.

### 2.4. Analysis of CT Images and BoAdy Composition

Body morphometry on CT was evaluated with an artificial intelligence software (AID-U^TM^, iAID Inc., Seoul, Korea), a fully automatic deep learning system for L3 selection and body composition assessment, as detailed in Ha et al.’s work [24]. The software uses a YOLOv3-based algorithm for automatic L3 inferior endplate level selection and a fully convolutional network (FCN) for segmentation of abdominal muscle and fat [23,25]. Selected L3-level CT images were automatically segmented to generate a boundary of total abdominal muscles, and the abdominal muscle and fat areas were measured. Next, experienced operators (Y.K and K.W.K) assessed the quality of the muscle segmentation in all images. The skeletal muscle area (SMA), including all muscles on the selected axial images (i.e., psoas, paraspinal, transversus abdominis, rectus abdominis, quadratus lumborum, and internal and external obliques) was demarcated using predetermined thresholds of −29 to +150 Hounsfield units. The visceral fat area (VFA) and the subcutaneous fat area (SFA) were also demarcated using fat tissue thresholds of −190 to −30 Hounsfield units.

### 2.5. Definition of Visceral Obesity and Sarcopenia

Visceral obesity was defined as a VFA >100 cm^2^ based on the Japan Society of the Study of Obesity guidelines [26]. The skeletal muscle index (SMI) was calculated as the SMA divided by the height squared in meters (cm^2^/m^2^). Sarcopenia was defined using a diagnostic cutoff based on a T-score of −2.0 in healthy Korean patients [27]. The number of individuals with sarcopenia was calculated as an SMI at L3 of <39.8 cm^2^/m^2^ in men and <28.4 cm^2^/m^2^ in women or as an SMA at L3 of <119.3 cm^2^ in men and <74.2 cm^2^ in women.

### 2.6. Statistical Analysis

Continuous variables are reported as mean ± standard deviation (SD) and categorical variables as numbers and percentages unless otherwise specified. VFA, SFA, SMA, and SMI before and after adrenalectomy were compared using paired *t*-tests, whereas the changes of catecholamine metabolites were assessed with the Wilcoxon signed-rank test. The prevalence of visceral obesity and sarcopenia based on SMA and SMI at baseline and follow-up was compared using McNemar’s test for paired proportions. To determine the associations of baseline catecholamine metabolites with changes in CT-determined body composition before and after adrenalectomy, multivariable linear regression analyses were performed after adjustment for age, sex, body mass index (BMI), and follow up duration. The associations between changes in catecholamine metabolites and changes in CT-determined body composition after surgery were also evaluated by multivariable linear regression analyses. A two-sided *p*-value of <0.05 was considered statistically significant. All statistical analyses were performed using SPSS for Windows version 21.0 (IBM Corp.: Armonk, NY, USA) and R version 4.1.0 (R Foundation for Statistical Computing, Vienna, Austria).

## 3. Results

The baseline characteristics of 313 patients with pheochromocytoma in the study are listed in Table 1. The mean age was 50.6 ± 13.6 years (range, 12–92), and 171 (54.6%) were women. Among the 313 patients, 20 (6.4%) and 29 (9.3%) had genetic mutations and metastatic or bilateral lesions, respectively. Laparoscopic surgery was performed on 215 (68.7%) patients, while the remaining patients underwent open surgery. A median follow-up duration for repeat CTs was 25 months (range, 3–48).

**Figure 1:**
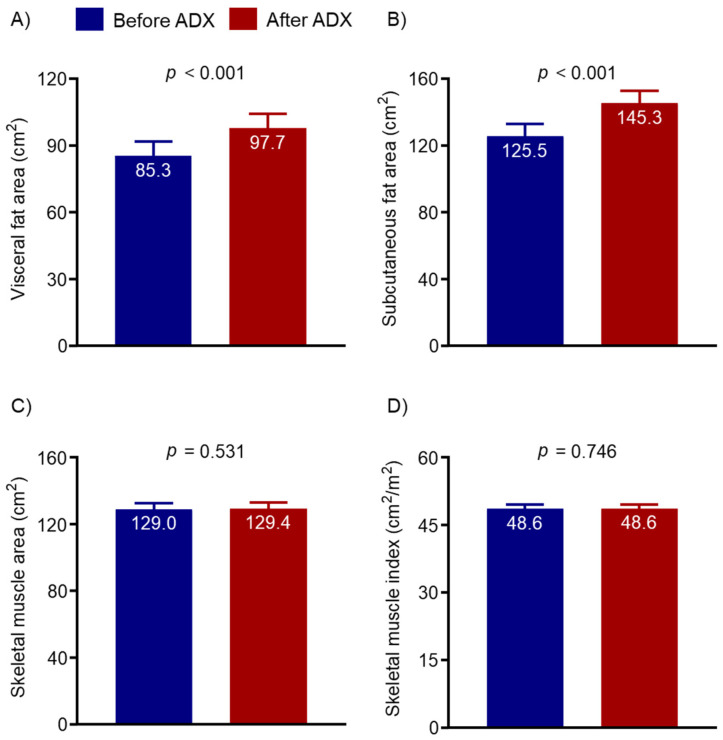
Changes in computed tomography-determined body compositions including visceral fat area (**A**), subcutaneous fat area (**B**), skeletal muscle area (**C**), and skeletal muscle index (**D**), before and after adrenalectomy in patients with pheochromocytoma. All *p*-values were calculated using the paired *t*-test or Wilcoxon signed-rank test, as appropriate. ADX, adrenalectomy.

**Figure 2:**
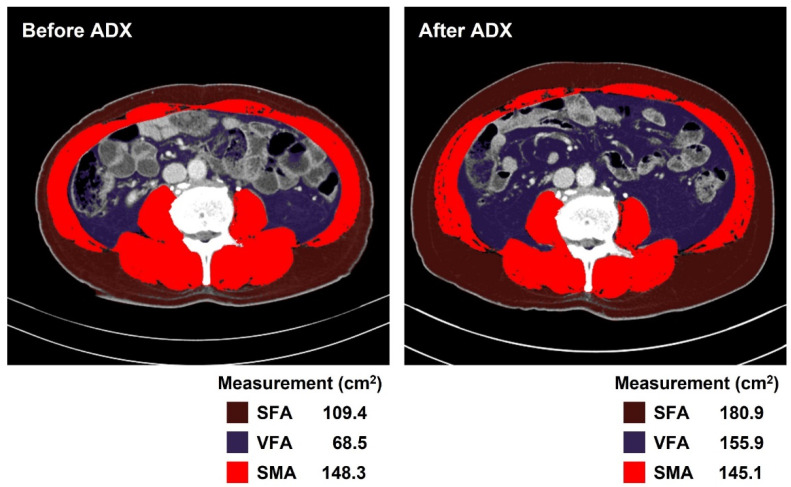
Representative image for body composition measurement using automated artificial intelligence software AID-U^TM^ (iAID inc, Seoul, Korea). Subcutaneous fat area (SFA; brown) and visceral fat area (VFA; purple) increased following adrenalectomy (ADX), whereas skeletal muscle area (SMA; red) statistically remained unchanged. These findings were observed in 266 out of 313 patients (85.0%).

Changes in catecholamine metabolites and CT-determined body composition before and after adrenalectomy were compared across all participants (Table 2 and Figure 1). As expected, metanephrine and normetanephrine levels in plasma and urine significantly decreased after adrenalectomy (all *p* < 0.001) and 96.2% (301 out of 313) had fractionated metanephrine levels in plasma and urine below the upper limit of the reference ranges. Notably, VFA and SFA significantly increased by 14.5% and 15.8%, respectively (both *p* < 0.001), after adrenalectomy. However, there was no difference in muscle parameters, including SMA and SMI, after surgery in patients with pheochromocytoma. Representative CT images measuring body composition are presented in Figure 2.

**Figure 3:**
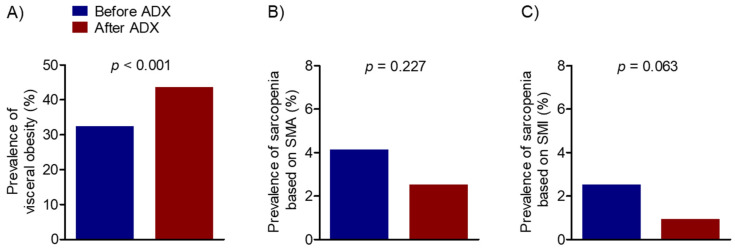
Changes in the prevalence of computed tomography-determined visceral obesity (**A**), sarcopenia based on skeletal muscle area (**B**), and skeletal muscle index (**C**) before and after adrenalectomy in patients with pheochromocytoma. All *p*-values were calculated using McNemar’s test for paired proportions. ADX, adrenalectomy; SMA, skeletal muscle area; SMI, skeletal muscle index.

The prevalence of CT-determined visceral obesity and sarcopenia was compared between the baseline and the median follow-up period of 25 months following adrenalectomy (Figure 3). Among the total 313 participants, those with visceral obesity significantly increased from 103 (32.9%) at baseline to 138 (44.1%) after surgery (*p* < 0.001). In contrast, there was no difference in the prevalence of sarcopenia based on SMA and SMI before and after adrenalectomy.

The associations of baseline catecholamine metabolites with changes in CT-based body composition following adrenalectomy were analyzed (Table 3). In an unadjusted model, higher levels of plasma metanephrine and urine normetanephrine at baseline were related to greater increases in SFA, and their statistical significance remained after adjusting for age, sex, BMI, and follow-up duration (*p* = 0.001 to 0.031). However, no baseline catecholamine metabolites were associated with changes in SMA and SMI in repeat CT scans, regardless of adjustment models.

We then examined the relationships between changes in catecholamine metabolites and CT-determined body composition before and after adrenalectomy in patients with pheochromocytoma (Table 4). In the unadjusted and multivariable-adjusted models, higher reductions of plasma metanephrine and urine normetanephrine following adrenalectomy were associated with greater increases in SFA (*p* = 0.001 to 0.040). Among muscle parameters, only a greater reduction of urine normetanephrine after adrenalectomy was associated with an increase in SMI after adjusting for confounding factors (*p* = 0.042).

## 4. Discussion

Pheochromocytoma is a rare neoplasm, probably occurring in less than 0.2 percent of patients with hypertension, and commonly produces one or more catecholamines [1,2]. Although much research on metabolic alterations in these patients has been conducted to investigate the effects of sympathetic overstimulation on human homeostasis [3,28,29], there has been a paucity of thorough clinical studies in terms of physical changes. The presented study on patients with histologically confirmed pheochromocytoma assessed changes in CT-based body composition after adrenalectomy and found that VFA and SFA, but not skeletal muscle parameters, were markedly higher after surgery. Similarly, the prevalence of visceral obesity significantly increased, whereas the prevalence of sarcopenia remained unchanged after adrenalectomy. To the best of our knowledge, this is the most extensive longitudinal study on body composition, including both fat and muscle, in patients with pheochromocytoma.

Experimental research suggests that SNS may influence fat metabolism through various mechanisms. Among three β-ARs present in human adipocytes, β_1_ and β_2_ are the most functionally active [11,12]. Catecholamines bound to these receptors sequentially activate adenylyl cyclase and cAMP-protein kinase A (PKA), then stimulate hormone-sensitive lipase, which degrades triglycerides to glycerol and fatty acids [12,30]. Activation of adipose triglyceride lipase may also contribute to catecholamine-induced lipolysis [31,32]. Furthermore, the β-AR-uncoupling protein 1 (UCP1) system regulates brown adipose tissue (BAT), which converts calories into heat in both rodents and humans [16,33], and BAT hyperactivity by catecholamine excess can lead to the burning of large amounts of fat via oxidation in mitochondria [15]. These data support the direct effects of sympathetic overstimulation on lipolysis. Among human studies, Okamura et al. [18] showed that fat mass in patients with pheochromocytoma was significantly increased after adrenalectomy. Although their study is among the first to implicate the importance of SNS on human adipose tissue, drawing finite conclusions from the data is challenging due to the small sample size of only 43 patients. We overcame this limitation in the present study by comparing body composition changes after surgery in a large cohort of more than 300 patients with pheochromocytoma and additionally reported the associations between baseline level, or delta change, of catecholamine metabolites and fat parameters. Consequently, we have provided more convincing evidence for lipolysis by sympathetic hyperactivity in humans.

The adipose tissue lining internal organs is called visceral fat, whereas that beneath the skin is termed subcutaneous fat. Interestingly, the degree of catecholamine-induced lipolysis and hormone-sensitive lipase activity in rats may differ according to its location [34]. The expression level of ARs also could vary according to the type of adipose tissue [35,36]. Therefore, sympathetic activity may have different effects on the metabolic processes for visceral and subcutaneous adipocytes via distinct ARs in humans. Indeed, our study showed that the increase in SFA (15.8%) after adrenalectomy was greater than that of VFA (14.5%) and that catecholamine metabolite levels were primarily related to SFA rather than VFA. Further research is necessary to elucidate how SNS influences visceral and subcutaneous fat through different mechanisms in humans.

Muscle metabolism appears to be affected by favorable and adverse catecholamine activity. In detail, β_2_-AR stimulation may promote skeletal muscle hypertrophy via the up-regulation of PKA or phosphoinositol 3-kinase (PI3K)-AKT signals and resultant muscle anabolic pathways [10,14,37]. Chronic activation of α-AR, on the other hand, may cause increased oxidative stress and decreased blood flow because of vasoconstriction, causing muscle wasting [10,13,38]. As a result, the involvement of catecholamines in muscle homeostasis is complex, and their net effects remain unclear, particularly in human muscle health. In this regard, one of the most notable findings of our study was that SMA, SMI and the prevalence of sarcopenia were not affected by adrenalectomy. Given that this is the first longitudinal study to investigate changes of muscle phenotypes in pheochromocytoma, current evidence cannot support the critical role of sympathetic activity, at least, in human skeletal muscle.

The current study has major strengths in its longitudinal design, comparing changes in body composition, including both fat and muscle using a well-validated deep learning based CT scan. Furthermore, we consecutively enrolled all patients with histologically confirmed pheochromocytoma or paraganglioma to minimize selection bias. Several potential limitations should be considered when interpreting our results despite these strengths. First, non-functional adrenal tumors do not require surgery unless they are large or suspected of malignancy. We therefore had no available controls and comparative analysis was not possible. Second, although we considered key confounders in the analyses, we cannot exclude the possibility that the observed findings were attributed to uncontrolled factors that affect body composition, such as exercise, serum 25-hydroxyvitamin D level, or medications. Third, the follow-up duration for repeat CT scans was considered in the multivariable analyses; nevertheless, its various timing, ranging from 3 to 48 months, may affect the results. Lastly, our study population was exclusively Korean, so we cannot determine the global applicability of these data.

## 5. Conclusions

We observed considerable elevation in CT-measured VFA and SFA, and the prevalence of visceral obesity after adrenalectomy in patients with pheochromocytoma. In contrast, there were no changes in SMA and SMI, or the prevalence of sarcopenia. Furthermore, we observed that the severity of catecholamine excess was associated with a higher increase, especially in SFA after surgery. These data provide important clinical evidence that sympathetic hyperactivity contributes to lipolysis in visceral and subcutaneous adipose tissues, whereas its impact on human skeletal muscle remains undetermined. These findings further suggest that when discussing postoperative expectations in patients with pheochromocytoma, it is necessary to sufficiently explain changes in not only metabolic but also physical aspects.

## Figures and Tables

**Table 1:** Baseline characteristics of study participants.

Variables	Participants (*n* = 313)	Variables	Participants (*n* = 313)
Age (years)	50.6 ± 13.6	Systolic BP (mmHg)	127.4 ± 19.2
Women, *n* (%)	171 (54.6)	Diastolic BP (mmHg)	79.0 ± 11.9
Height (cm)	162.2 ± 8.4	Calcium (mg/dL)	9.1 ± 0.5
Weight (kg)	63.4 ± 11.4	Phosphorus (mg/dL)	3.8 ± 0.7
Body mass index (kg/m^2^)	24.0 ± 3.5	Glucose (mg/dL)	118.5 ± 36.5
Genetic mutations, *n* (%)	20 (6.4)	HbA1c (%)	6.1 ± 0.9
Metastatic or bilateral lesion, n (%)	29 (9.3)	Creatinine (mg/dL)	0.79 ± 0.19
Type of operation, *n* (%)		Total cholesterol (mg/dL)	184.0 ± 37.3
Laparoscopic	215 (68.7)	AST (IU/L)	21.9 ± 8.1
Open	98 (31.3)	ALT (IU/L)	22.3 ± 14.2

Continuous variables are reported as mean ± standard deviation and categorical variables as numbers and percentages. ALT, alanine aminotransferase; AST, aspartate aminotransferase; BP, blood pressure; HbA1c, glycated hemoglobin.

**Table 2:** Changes in computed tomography-determined body composition and catecholamine metabolites before and after adrenalectomy in patients with pheochromocytoma.

Variables	Before ADX	After ADX	*p*
Catecholamine metabolites			
Plasma metanephrine (nmol/L), median (IQR)	**0.44 (0.18–2.26)**	**0.10 (0.08–0.13)**	**<0.001**
Plasma normetanephrine (nmol/L), median (IQR)	**3.64 (1.56–9.46)**	**0.50 (0.37–0.63)**	**<0.001**
Urine metanephrine (μg/day), median (IQR)	**355.5 (110.5–1456.2)**	**45.5 (29.5–70.8)**	**<0.001**
Urine normetanephrine (μg/day), median (IQR)	**1180.4 (564.2–2673.6)**	**178.7 (128.2–232.0)**	**<0.001**
VFA (cm_2_)	**85.3 ± 57.4**	**97.7 ± 59.4**	**<0.001**
SFA (cm_2_)	**125.5 ± 67.1**	**145.3 ± 65.9**	**<0.001**
SMA (cm_2_)	129.0 ± 31.6	129.4 ± 31.5	0.531
SMI (cm_2_/m_2_)	48.6 ± 9.1	48.6 ± 8.9	0.746

All *p*-values were calculated using the paired *t*-test or Wilcoxon signed-rank test, as appropriate. **Bold** means that values are statistically significant. ADX, adrenalectomy; IQR, interquartile range; VFA, visceral fat area; SFA, subcutaneous fat area; SMA, skeletal muscle area; SMI, skeletal muscle index.

**Table 3:** Association of baseline catecholamine metabolites with changes in computed tomography-determined body composition before and after adrenalectomy in patients with pheochromocytoma.

Independent Variable: Plasma Metanephrine						
Change (Baseline to Follow-Up)	Unadjusted		Age and Sex-Adjusted		Multivariable-Adjusted	
	β (95% CI)	*p*	β (95% CI)	*p*	β (95% CIs	*p*
ΔVFA (cm^2^)	0.699 (−0.364 to 1.761)	0.196	0.714 (−0.351 to 1.780)	0.188	0.641 (−0.414 to 1.697)	0.232
ΔSFA (cm^2^)	**1.339** **(0.121 to 2.558)**	**0.031**	**1.471** **(0.217 to 2.726)**	**0.022**	**1.364** **(0.134 to 2.594)**	**0.030**
ΔSMA (cm^2^)	−0.070 (−0.398 to 0.259)	0.677	−0.057 (−0.395 to −0.281)	0.738	−0.084 (−0.418 to 0.250)	0.619
ΔSMI (cm^2^/m^2^)	−0.014 (−0.133 to 0.105)	0.822	−0.015 (−0.137 to 0.108)	0.813	−0.023 (−0.145 to 0.100)	0.716
**Independent Variable: Plasma Normetanephrine**						
**Change** **(Baseline to Follow-Up)**	**Unadjusted**		**Age and Sex-Adjusted**		**Multivariable-Adjusted**	
	**β (95% CI)**	***p***	**β (95% CI)**	***p***	**β (95% CI)**	***p***
ΔVFA (cm^2^)	0.278 (−0.124 to 0.680)	0.175	0.307 (−0.087 to 0.701)	0.126	0.266 (−0.124 to 0.656)	0.180
ΔSFA (cm^2^)	0.406 (−0.057 to 0.870)	0.085	0.407 (−0.060 to 0.875)	0.087	0.347 (−0.111 to 0.805)	0.137
ΔSMA (cm^2^)	0.114 (−0.009 to 0.237)	0.069	0.116 (−0.008 to 0.240)	0.067	0.103 (−0.020 to 0.225)	0.100
ΔSMI (cm^2^/m^2^)	0.047 (0.002 to 0.091)	0.040	0.048 (0.003 to 0.093)	0.036	0.044 (−0.001 to 0.089)	0.052
**Independent Variable: Urine Metanephrine**						
**Change** **(Baseline to Follow-Up)**	**Unadjusted**		**Age and Sex-Adjusted**		**Multivariable-Adjusted**	
	**β (95% CI)**	***p***	**β (95% CI)**	***p***	**β (95% CI)**	***p***
ΔVFA (cm^2^)	0.046 (−0.092 to 0.184)	0.516	0.041 (−0.095 to 0.176)	0.556	0.041 (−0.092 to 0.175)	0.543
ΔSFA (cm^2^)	0.084 (−0.070 to 0.237)	0.282	0.087 (−0.069 to 0.242)	0.274	0.083 (−0.070 to 0.236)	0.284
ΔSMA (cm^2^)	−0.021 (−0.065 to 0.023)	0.355	−0.019 (−0.063 to 0.025)	0.401	−0.021 (−0.064 to 0.022)	0.328
ΔSMI (cm^2^/m^2^)	−0.006 (−0.022 to 0.010)	0.463	−0.006 (−0.022 to 0.010)	0.494	−0.007 (−0.022 to 0.009)	0.414
**Independent Variable: Urine Normetanephrine**						
**Change** **(Baseline to Follow-Up)**	**Unadjusted**		**Age and Sex-Adjusted**		**Multivariable-Adjusted**	
	**β (95% CI)**	***p***	**β (95% CI)**	***p***	**β (95% CI)**	***p***
ΔVFA (cm^2^)	0.148 (−0.001 to 0.297)	0.052	0.143 (−0.006 to 0.292)	0.059	0.122 (−0.026 to 0.271)	0.106
ΔSFA (cm^2^)	**0.290** **(0.128 to 0.453)**	**0.001**	**0.300** **(0.132 to 0.467)**	**0.001**	**0.291** **(0.125 to 0.458)**	**0.001**
ΔSMA (cm^2^)	0.047 (−0.001 to 0.095)	0.053	0.040 (−0.009 to 0.088)	0.106	0.041 (−0.007 to 0.089)	0.091
ΔSMI (cm^2^/m^2^)	0.019 (0.001 to 0.036)	0.035	0.017 (−0.001 to 0.034)	0.063	0.017 (0 to 0.035)	0.050

All *p*-values were calculated using multivariable linear regression analyses. The multivariable adjustment model includes age, sex, body mass index, and follow-up duration. **Bold** indicates values are statistically significant. CI, confidence interval; VFA, visceral fat area; SFA, subcutaneous fat area; SMA, skeletal muscle area; SMI, skeletal muscle index.

**Table 4:** Association between changes in catecholamine metabolites and changes in computed tomography-determined body composition before and after adrenalectomy in patients with pheochromocytoma.

Independent Variable: ΔPlasma Metanephrine (Baseline to Follow-Up)						
Change (Baseline to Follow-Up)	Unadjusted		Age and Sex-Adjusted		Multivariable-Adjusted	
	β (95% CI)	*p*	β (95% CI)	*p*	β (95% CI)	*p*
ΔVFA (cm^2^)	−0.740 (−1.832 to 0.353)	0.183	−0.766 (−1.871 to 0.340)	0.173	−0.658 (−1.755 to 0.440)	0.238
ΔSFA (cm^2^)	**−1.352** **(−2.614 to −0.090)**	**0.036**	**−1.498** **(−2.805 to −0.190)**	**0.025**	**−1.343** **(−2.626 to −0.060)**	**0.040**
ΔSMA (cm^2^)	0.072 (−0.265 to 0.409)	0.675	0.062 (−0.287 to 0.411)	0.726	0.098 (−0.247 to 0.443)	0.575
ΔSMI (cm^2^/m^2^)	0.017 (−0.105 to 0.139)	0.788	0.019 (−0.107 to 0.146)	0.763	0.030 (−0.095 to 0.156)	0.633
**Independent Variable: ΔPlasma Normetanephrine (Baseline to Follow-up)**						
**Change** **(Baseline to Follow-Up)**	**Unadjusted**		**Age and Sex-Adjusted**		**Multivariable-Adjusted**	
	**β (95% CI)**	***p***	**β (95% CI)**	***p***	**β (95% CI)**	***p***
ΔVFA (cm^2^)	−0.302 (−0.716 to 0.113)	0.152	−0.322 (−0.729 to 0.085)	0.120	−0.276 (−0.680 to 0.129)	0.180
ΔSFA (cm^2^)	−0.433 (−0.914 to 0.048)	0.077	−0.431 (−0.917 to 0.054)	0.081	−0.361 (−0.837 to 0.115)	0.136
ΔSMA (cm^2^)	−0.112 (−0.238 to 0.015)	0.084	−0.112 (−0.240 to 0.015)	0.084	−0.097 (−0.224 to 0.029)	0.131
ΔSMI (cm^2^/m^2^)	−0.044 (−0.090 to 0.001)	0.058	−0.045 (−0.091 to 0.001)	0.054	−0.041 (−0.087 to 0.005)	0.081
**Independent Variable: ΔUrine Metanephrine (Baseline to Follow-Up)**						
**Change** **(Baseline to Follow-Up)**	**Unadjusted**		**Age and Sex-Adjusted**		**Multivariable-Adjusted**	
	**β (95% CI)**	***p***	**β (95% CI)**	***p***	**β (95% CI)**	***p***
ΔVFA (cm^2^)	0.006 (−0.150 to 0.162)	0.941	0.0004 (−0.154 to 0.155)	0.995	−0.005 (−0.157 to 0.148)	0.953
ΔSFA (cm^2^)	−0.026 (−0.204 to 0.152)	0.773	−0.027 (−0.207 to 0.153)	0.767	−0.031 (−0.207 to 0.145)	0.727
ΔSMA (cm^2^)	0.027 (−0.024 to 0.078)	0.296	0.024 (−0.026 to 0.075)	0.347	0.024 (−0.025 to 0.074)	0.337
ΔSMI (cm^2^/m^2^)	0.008 (−0.010 to 0.027)	0.383	0.007 (−0.011 to 0.026)	0.435	0.007 (−0.011 to 0.025)	0.423
**Independent Variable: ΔUrine Normetanephrine (Baseline to Follow-Up)**						
**Change** **(Baseline to Follow-Up)**	**Unadjusted**		**Age and Sex-Adjusted**		**Multivariable-Adjusted**	
	**β (95% CI)**	***p***	**β (95% CI)**	***p***	**β (95% CI)**	***p***
ΔVFA (cm^2^)	−0.128 (−0.287 to 0.031)	0.113	−0.124 (−0.285 to 0.038)	0.132	−0.108 (−0.270 to 0.053)	0.187
ΔSFA (cm^2^)	**−0.311** **(−0.488 to −0.134)**	**0.001**	**−0.327** **(−0.509 to −0.145)**	**0.001**	**−0.319** **(−0.499 to −0.139)**	**0.001**
ΔSMA (cm^2^)	−0.052 (−0.104 to 0.001)	0.053	−0.047 (−0.100 to 0.006)	0.080	−0.047 (−0.100 to 0.005)	0.075
ΔSMI (cm^2^/m^2^)	**−0.021** **(−0.040 to −0.002)**	**0.034**	**−0.019** **(−0.039 to −0.0002)**	**0.048**	**−0.020** **(−0.039 to −0.001)**	**0.042**

All *p*-values were calculated using multivariable linear regression analyses. The multivariable adjustment model includes age, sex, body mass index, and follow-up duration. **Bold** indicates values are statistically significant. CI, confidence interval; VFA, visceral fat area; SFA, subcutaneous fat area; SMA, skeletal muscle area; SMI, skeletal muscle index.

## Data Availability

The datasets analyzed during the current study are available from the corresponding author on reasonable request.
